# The In Vitro Anti-Parasitic Activities of Emodin toward *Toxoplasma gondii*

**DOI:** 10.3390/ph16030447

**Published:** 2023-03-16

**Authors:** Oluyomi Stephen Adeyemi, Kosei Ishii, Kentaro Kato

**Affiliations:** 1 Laboratory of Sustainable Animal Environment, Graduate School of Agricultural Science, Tohoku University, 232-3 Yomogida, Naruko-onsen, Osaki 989-6711, Miyagi, Japan; 2 Medicinal Biochemistry and Toxicology Laboratory, Department of Biochemistry, Landmark University, Omu-Aran 251101, Kwara State, Nigeria

**Keywords:** drug discovery, medicinal biochemistry, phytomedicine, toxoplasmosis

## Abstract

Currently, toxoplasmosis affects nearly one-third of the world’s population, but the available treatments have several limitations. This factor underscores the search for better therapy for toxoplasmosis. Therefore, in the current investigation, we investigated the potential of emodin as a new anti-*Toxoplasma gondii* while exploring its anti-parasitic mechanism of action. We explored the mechanisms of action of emodin in the presence and absence of an in vitro model of experimental toxoplasmosis. Emodin showed strong anti-*T. gondii* action with an EC_50_ value of 0.03 µg/mL; at this same effective anti-parasite concentration, emodin showed no appreciable host cytotoxicity. Likewise, emodin showed a promising anti-*T. gondii* specificity with a selectivity index (SI) of 276. Pyrimethamine, a standard drug for toxoplasmosis, had an SI of 2.3. The results collectively imply that parasite damage was selective rather than as a result of a broad cytotoxic effect. Furthermore, our data confirm that emodin-induced parasite growth suppression stems from parasite targets and not host targets, and indicate that the anti-parasite action of emodin precludes oxidative stress and ROS production. Emodin likely mediates parasite growth suppression through means other than oxidative stress, ROS production, or mitochondrial toxicity. Collectively, our findings support the potential of emodin as a promising and novel anti-parasitic agent that warrants further investigation.

## 1. Introduction

The parasitic disease toxoplasmosis poses a global health threat [1,2]. Toxoplasmosis, which currently affects nearly one-third of humans, is caused by *Toxoplasma gondii*, an intracellular, obligate parasite with a low host specificity [3,4]. In healthy individuals, *Toxoplasma* infection may go unnoticed, but in immunocompromised or pregnant individuals, it may result in morbidity and mortality [5]. Consuming tissue cysts (which contain the bradyzoite form) in undercooked meat or oocysts (which contain the sporozoite form) discharged in felid fecal material can lead to host infection [6]. The parasite develops into tachyzoites, which quickly infect the host’s tissues, when an intermediate host contracts *T. gondii* infection. Typically, the immune system and medications can clear active *Toxoplasma* infection, but occasionally, tachyzoites evade clearance and transform into bradyzoites, resulting in cyst formation in infected tissues. The relatively inactive tissue cysts can result in recurrent infections, especially in people who have compromised immune systems. Sulfadiazine and/or pyrimethamine are the currently available therapeutic options for toxoplasmosis, but these medications have poor effectiveness and the tolerance for them is low [5]. Furthermore, the available treatments do not eliminate tissue cysts. Therefore, there is a need for improved medicines and/or approaches to treating toxoplasmosis.

**Figure 1:**
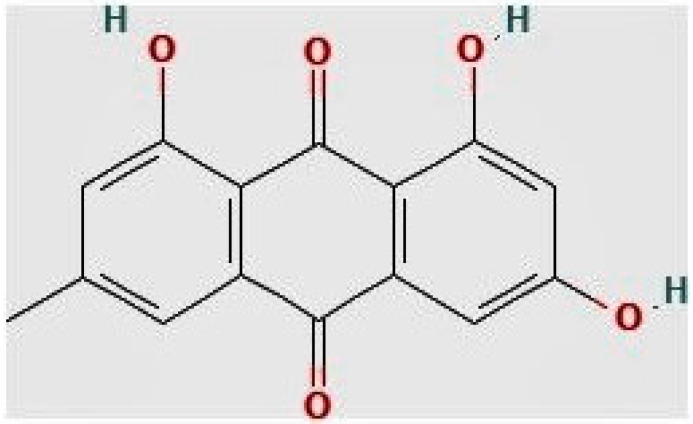
Chemical structure of emodin Source: https://pubchem.ncbi.nlm.nih.gov/#query=emodin (accessed on 15 December 2022).

The emerging evidence indicates that testing a variety of substances for possible anti-parasitic action is a viable strategy for identifying new treatment options for infectious diseases [1,7]. Recently, we screened chemical libraries containing 1143 compounds and discovered 32 “hit” compounds that inhibited *T. gondii* growth in vitro [6]. One of these hit compounds is a naturally occurring anthraquinone derivative known as emodin (1,3,8-trihydroxy-6-methylanthraquinone) (Figure 1). Emodin is an active component of several Chinese herbs, including *Rheum palmatum*, *Polygonum cuspidatum*, *Polygonum multiflorum*, *and Aloe vera* [8]. It has been shown in numerous studies to possess antibacterial [9], anticancer [10,11], antifungal [12], anti-inflammatory [13], and antioxidant [14,15] properties. Other studies have reported the antiprotozoal [16,17] and neuroprotective [18,19] properties of emodin. Additionally, emodin and its derivatives are useful as stimulant laxatives for treating constipation [17,20]. However, it is also important to identify the mechanism of action of potential therapeutic compounds. While phenotypic screening can identify new candidates for drug development, understanding their mechanisms of action enhances their prospects for rational design and development. Therefore, knowledge of how these anti-parasitic molecules work in a parasite is important to improve and optimize their potential. This is because identifying the target(s) for potential lead compounds can assist rational chemistry in terms of improving the selective binding of drugs to parasite targets and ensuring that structural modifications to improve the pharmacokinetic and toxicity profiles do not compromise activity. Currently, the action mechanisms of emodin as an anti-*Toxoplasma* agent is unclear. Accordingly, in the current study, we sought to explore the likely anti-parasitic mechanism of action of emodin in an in vitro model of experimental toxoplasmosis. To our knowledge, our study is the first report on the action mechanism of emodin as an anti-*Toxoplasma* agent and we provide evidence to support that emodin’s anti-parasitic action does not involve the production of reactive oxygen species (ROS), oxidative stress, and/or mitochondrial toxicity.

## 2. Results

### 2.1. Emodin Significantly Restricts Parasite Growth

**Figure 2:**
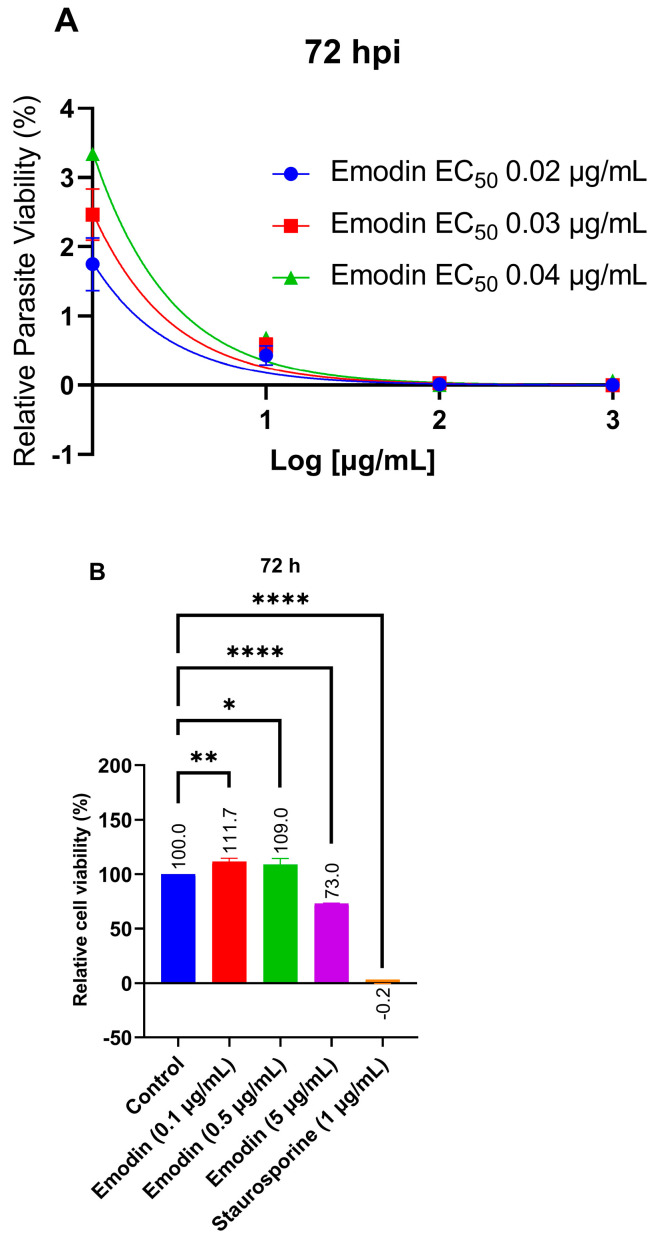
(**A**) Effect of emodin on parasite viability after a 72 h treatment. Dose–response curve showing the anti-parasite activities of emodin. The parasite growth curve was determined using a luciferase reporter assay after a 72 h incubation. (**B**) Host cell viability. In the absence of *T. gondii* infection, the host monolayers were treated with emodin at ten times the EC_50_ for *T. gondii*, and cell viability was determined after a 72-h incubation. The data are means ± standard deviation (SD). The experiment was performed in triplicate and repeated three times independently. ns—not significant at *p* > 0.05. *—significant at *p* < 0.05, ** at *p* < 0.01, and **** at *p* < 0.0001.

Previously, we reported our findings on a group of natural molecules, which included emodin that caused strong and selective in vitro inhibition of *T. gondii* growth. To confirm this earlier report [6], we assessed emodin at various concentrations (between 0.0 and 1000 µg/mL) for an anti-parasitic efficacy and host cell cytotoxic potential. We added emodin to a freshly purified parasite suspension to a growing human foreskin fibroblast (HFF ATCC^®^) cell to assess its anti-*T. gondii* activity. After 72 h, we determined the parasite viability by using a luminescence-based assay. Emodin dose-dependently suppressed the parasite growth in vitro by >95% (Figure 2A). The finding confirms the ability of emodin to restrict *T. gondii* growth in vitro. Emodin had an EC_50_ value of 0.03 µg/mL (Table 1) toward the parasite. To validate our screening assay, we used a reference drug (pyrimethamine) as a positive control. As expected, pyrimethamine decreased the parasite viability (data not shown).

We did not observe any host toxicity when we assessed the cytotoxicity of emodin toward the host. The estimated host cell IC_50_ was 8.3 µg/mL (Table 1). Even at higher doses, emodin caused no significant host cell toxicity (Figure 2B). Furthermore, when we estimated the selectivity index (SI, ratio of toxicity to parasite versus host cell), we found that emodin showed a strong selective toxicity toward the parasite versus the host cell by about 276-fold. By contrast, the SI for pyrimethamine was 2.6. Therefore, for subsequent experiments, we tested emodin at the EC_50_ or EC_90_ doses unless otherwise stated.

**Figure 3:**
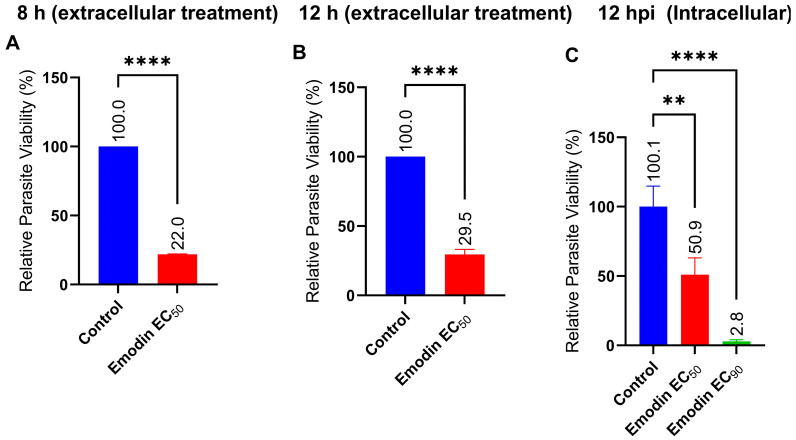
Effect of emodin on parasite growth under extracellular or intracellular conditions. (**A**) Extracellular at 8 h. (**B**) Extracellular at 12 h. (**C**) Intracellular at 12 hpi (hour post-infection). Growth inhibition was determined using a luciferase reporter assay. The data are means ± standard deviation (SD). The experiment was performed in triplicate and repeated three times independently. ns—not significant at *p* > 0.05. **—significant at *p* < 0.01 and **** at *p* < 0.0001. EC_50_—0.03 µg/mL and EC_90_—0.05 µg/mL.

To assess whether emodin has a direct effect on parasites, we incubated freshly purified parasites with emodin for 8 or 12 h extracellularly. Interestingly, emodin drastically reduced the parasite growth relative to the control (Figure 3A,B). In parallel, after a 12-h incubation under the same conditions, emodin caused a significant (*p* < 0.05) reduction in the parasite growth intracellularly compared with the control (Figure 3C). Together, these data demonstrate direct anti-parasite effects and suggest that emodin has parasite targets.

**Figure 4:**
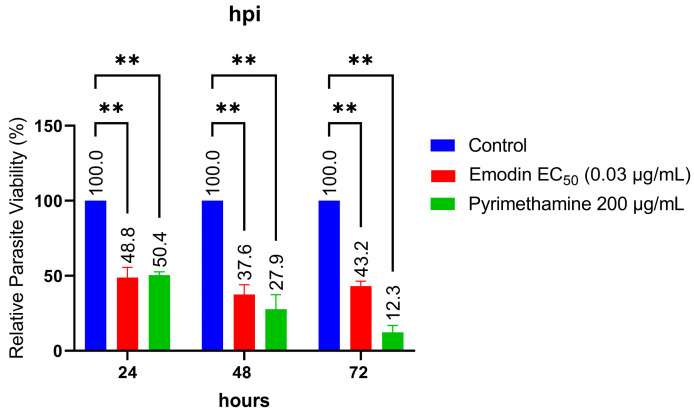
Effect of emodin on parasite growth at different timepoints. Growth inhibition was determined using a luciferase reporter assay at 24, 48, and 72 h. The data are means ± standard deviation (SD). The experiment was performed in triplicates and repeated three times independently. **—significant at *p* < 0.01.

Next, we sought to determine the time kinetics of the parasite growth suppression caused by emodin. Emodin did not cause a distinct time-dependent suppression of parasite growth when incubated with freshly lysed parasites in host monolayers for different timepoints of 24, 48, and 72 h. (Figure 4). The results showed that the anti-parasitic effects of emodin peaked within the first 24 h of treatment. However, pyrimethamine showed a time-dependent effect with a gradual increase in parasite inhibition over 72 h.

**Figure 5:**
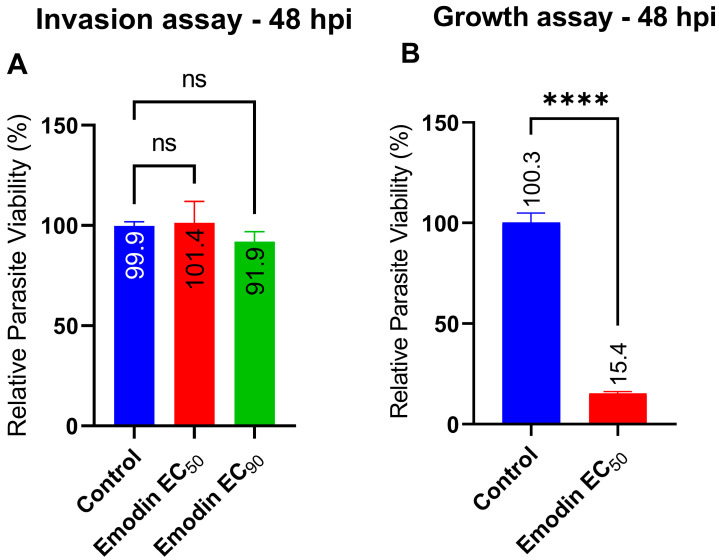
Effect of emodin on parasite invasion versus growth. (**A**) Invading parasites were treated with emodin for 1 h after which the medium was washed off to remove non-invading parasites. New culture medium was added and assay plates were incubated for 48 h. (**B**) In parallel, freshly purified parasites and emodin were added to growing host monolayers and incubated for 48 h. Parasite viability was measured using a luciferase reporter assay at 48 h. The data are means ± standard deviation (SD). The experiment was performed in triplicates and repeated three times independently. ns—not significant at *p* > 0.05. ****—significant at *p* < 0.0001. EC_50_—0.03 µg/mL; EC_90_—0.05 µg/mL.

We also checked whether emodin treatment affected parasite invasion. We found that emodin did not impede the capacity of the parasite to invade the host cells (Figure 5A); however, at the same EC_50_ concentrations and under the same conditions, emodin suppressed *T. gondii* growth (Figure 5B). Collectively, these findings suggest that emodin does not block parasite invasion of host cells.

### 2.2. Emodin Mildly Affects Parasite Infectivity but Has no Detectable Effect on Recovery

**Figure 6:**
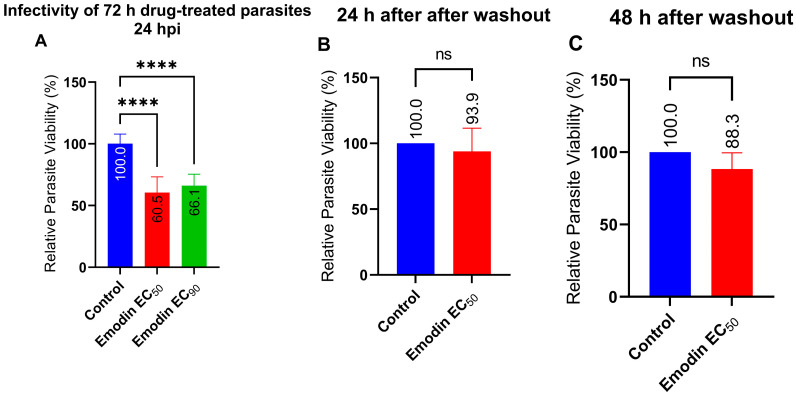
(**A**) Effect of emodin on parasite infectivity. Parasite infectivity was determined after a 72-h intracellular treatment. The treated parasites were harvested from the host, syringe released, and purified. Fresh host cells were then infected using the freshly purified parasites and growth was determined after a 24-h incubation. (**B**,**C**) Reversibility of treatment effects at 24 or 48 h, respectively. Invading parasites were treated with emodin for 24 hpi, after which the medium was removed, the cells were washed thoroughly, and the medium was replaced. The cells were incubated for 24 or 48 h after which the reversibility of treatment was evaluated through parasite viability. The data are means ± standard deviation (SD). The experiment was performed in triplicate and repeated three times independently. ns—not significant at *p* > 0.05. ****—significant at *p* < 0.0001. EC_50_—0.03 µg/mL; EC_90_—0.05 µg/mL.

Next, we sought to assess the effect of emodin on the ability of treated parasites to infect new host monolayers. In this experiment, we treated freshly purified parasites in host cells with emodin. After a 72-h incubation, we harvested the parasites and used them to infect fresh host cells. After a 24-h incubation, we determined parasite viability. Emodin mildly reduced the parasite infectivity compared with the control (Figure 6A). At both the EC_50_ and EC_90_ (0.03 and 0.05 µg/mL, respectively), the parasites retained a level of infectivity, although it was appreciably (*p* < 0.05) reduced compared with the control. These findings indicated that the anti-parasitic effect of emodin may be reversible following treatment removal and washout. Therefore, we assessed whether the anti-parasitic action of emodin was indeed reversible. We treated host monolayers inoculated with freshly purified parasites with emodin for 24 or 48 h. At each timepoint, we removed the treatment, washed the monolayers thoroughly, and replaced the medium with fresh medium. After a 24 h-incubation, we determined the parasite viability. The anti-parasitic action of emodin at both timepoints was reversible as the parasite growth slowly recovered (Figure 6B,C). Collectively, these findings suggest that the anti-parasitic effect of emodin is reversible and that the degree of reversibility is not influenced by the length of time the parasite is exposed to emodin. In addition, the capacity of the parasite to recover after treatment removal and washout may be related to the lack of impact of emodin on parasite infectivity.

### 2.3. Parasite Growth Is Unaffected following Pre-Treatment of Host Cells with Emodin

**Figure 7:**
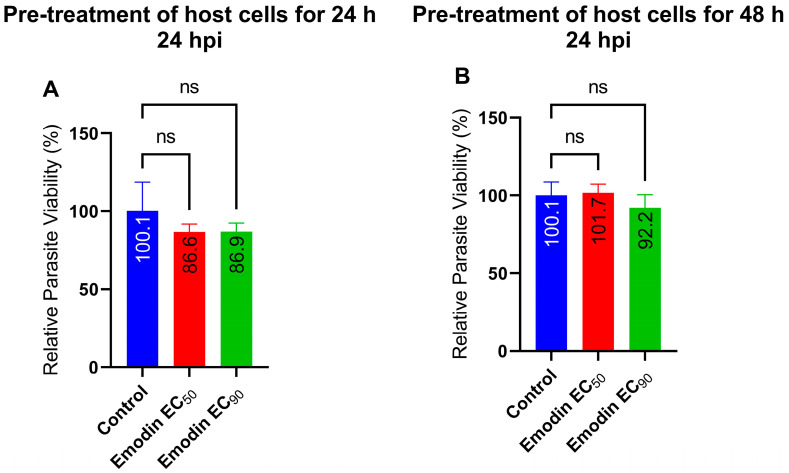
(**A**,**B**) Growing HFF monolayers were pre-treated with emodin for 24 or 48 h, respectively. The pre-treated host cells were washed thoroughly with fresh medium and freshly purified parasites were then allowed to invade and infect the pre-treated HFF monolayers. The parasite growth potential was determined after 24 h. The data are means ± standard deviation (SD). The experiment was performed in triplicate and repeated three times independently. ns—not significant at *p* > 0.05. EC_50_—0.03 µg/mL; EC_90_—0.05 µg/mL.

Next, we sought to assess whether the effect of emodin on the parasite viability was through a host target by pre-treating host cell monolayers with emodin. After a 24 or 48 h incubation, we removed the emodin, washed the monolayers thoroughly, and allowed the freshly lysed and purified parasites to infect the pre-treated host cells. After a 24-h incubation, we determined the parasite’s viability. Emodin pre-treatment of the host cells caused no appreciable reduction in the parasite growth compared to the mock pre-treated cells (Figure 7). This finding implies that anti-parasite action of emodin may not be dependent on host cell targets.

### 2.4. Trolox and L-Tryptophan Do Not Relieve Emodin-Induced Parasite Growth Suppression

**Figure 8:**
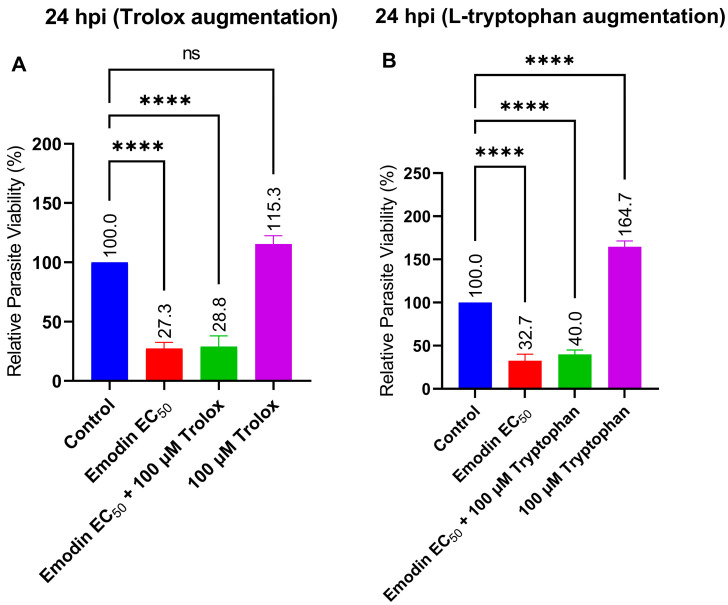
(**A**) Effect of Trolox augmentation on the anti-*Toxoplasma gondii* activity of emodin. (**B**) Effect of l-tryptophan augmentation on the anti-*T. gondii* activity of emodin. *T. gondii*-infected HFF monolayers were co-treated with emodin and Trolox or l-tryptophan at the indicated concentration and parasite viability was determined after a 24-h incubation. The data are means ± standard deviation (SD). The experiment was repeated three times independently. ns—not significant at *p* > 0.05. ****—significant at *p* < 0.0001. EC_50_—0.03 µg/mL.

In this experiment, we sought to determine the mode of anti-parasitic action. First, we explored whether oxidative stress or reactive oxygen species (ROS) could be involved in the anti-parasite action of emodin. We added the antioxidant Trolox to our anti-parasitic screening experiment. Trolox did not affect the anti-parasitic action of emodin (Figure 8A). Parasite inhibition did not improve in the presence of Trolox. The results suggest that ROS or oxidative stress are not involved in the anti-parasitic activity of emodin.

Since *T. gondii* are auxotroph for l-tryptophan, we sought to determine whether the addition of this amino acid would modulate the anti-parasitic action of emodin. Therefore, we included l-tryptophan in the anti-parasitic screening experiment. L-tryptophan addition did not reduce the suppressive effect of emodin on the parasite growth (Figure 8B). The parasite inhibition did not improve with the addition of l-tryptophan. Together, the data indicate that l-tryptophan augmentation is insufficient to relieve the parasite growth suppression caused by emodin. These findings also support the lack of host target involvement in the anti-parasitic action of emodin; *T. gondii* acquire their tryptophan from the host environment.

### 2.5. Emodin Does Not Modify Redox Balance in the Absence or Presence of Toxoplasma Infection

**Figure 9:**
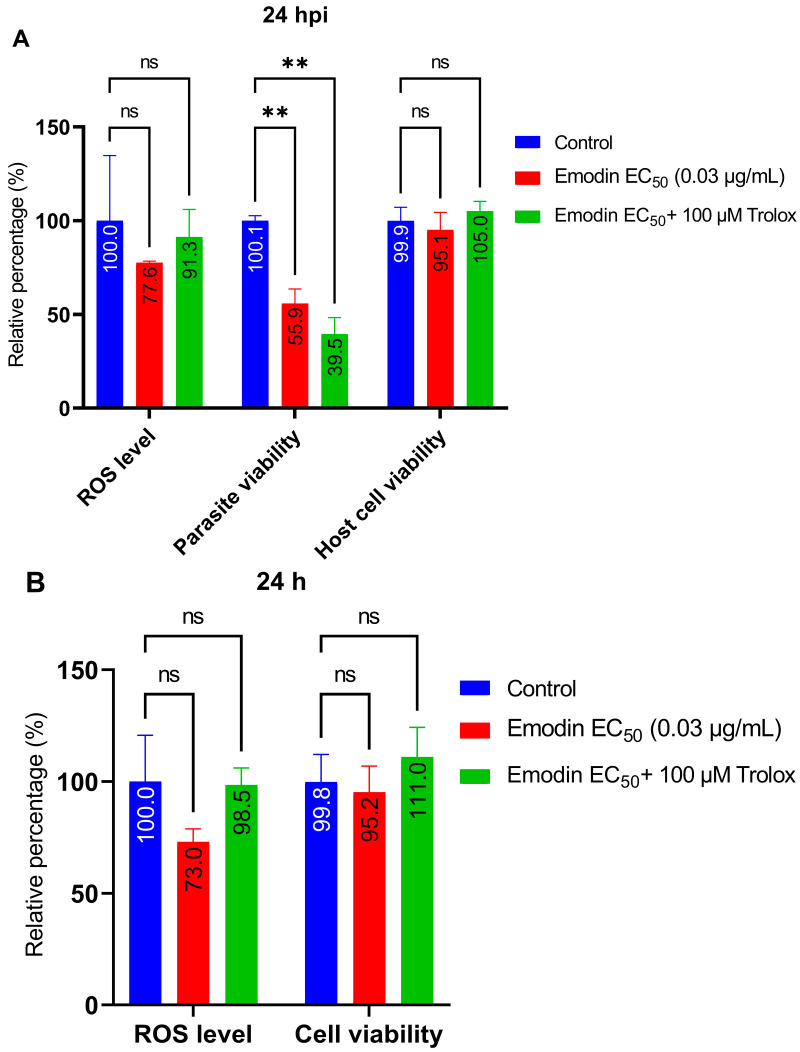
(**A**,**B**) Effect of emodin on redox status by measuring intracellular reactive oxygen species (ROS) production in the absence and presence of *Toxoplasma* infection. The infected or un-infected HFF monolayers were treated with a combination of emodin and Trolox. The relative ROS level was determined after a 24-h incubation. The parasite viability and host cell toxicity were also determined from the same assay plates by multiplexing. ROS determination was accomplished using a Promega ROS-Glo™ H_2_O_2_ assay (Promega). The data are means ± standard deviation (SD). The experiment was repeated three times independently. ns—not significant at *p* > 0.05. **—significant at *p* < 0.01.

Here, we sought to determine whether exposure to emodin causes ROS production. To assess the intracellular ROS levels, we used a commercial assay kit (ROS-Glo™ H_2_O_2_, Promega, Madison, WI, USA). Emodin did not influence the ROS generation, but rather, it reduced the global level of intracellular ROS relative to the control, albeit not significantly (Figure 9A,B). We matched the ROS level with parasite and cell viability upon emodin treatment and found that, while emodin reduced parasite viability, the host cells remained unaffected. In contrast, emodin reduced the ROS level compared with the control (Figure 9A). Interestingly, we observed similar effects in the absence of the *Toxoplasma* infection (Figure 9B). Collectively, these findings strengthen the concept that the anti-parasite action of emodin does not involve ROS or oxidative stress.

**Figure 10:**
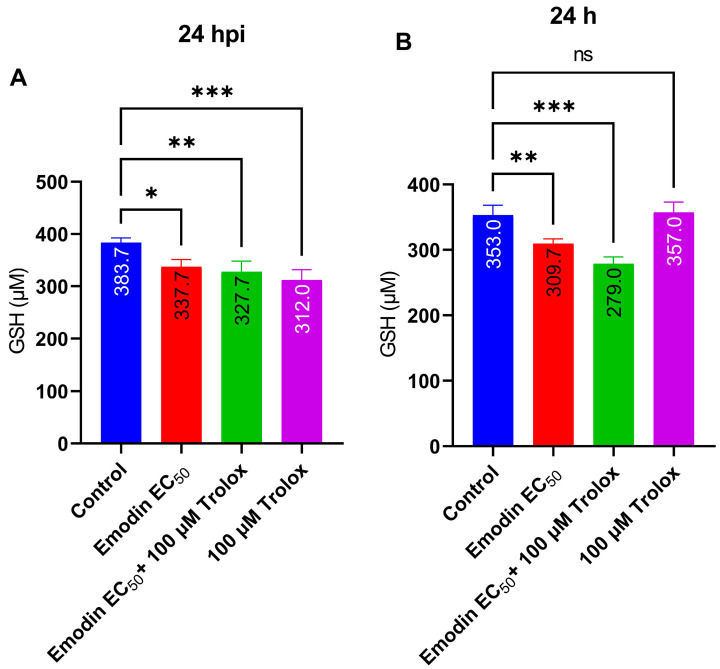
(**A**,**B**) Effect of emodin on levels of reduced glutathione (GSH) in the absence and presence of *Toxoplasma* infection. The infected or un-infected HFF monolayers were treated with a combination of NP and Trolox. The relative GSH level was determined after a 24-h incubation using a GSH-Glo™ glutathione assay kit (Promega). The data are means ± standard deviation (SD). The experiment was performed in triplicates and repeated three times independently. ns—not significant at *p* > 0.05. *—significant at *p* < 0.05, ** at *p* < 0.01, and *** at *p* < 0.001. EC_50_—0.03 µg/mL.

We also determined the intracellular GSH levels in the absence and presence of the *Toxoplasma* infection. Emodin did not affect GSH in the absence or presence of the *Toxoplasma* infection (Figure 10A,B). However, Trolox potentiated the capacity of emodin to reduce the GSH level in the absence of the *T. gondii* infection.

### 2.6. Mitochondria Membrane Integrity and ATP Production in the Absence or Presence of Toxoplasma Infection Are Not Affected by Emodin

**Figure 11:**
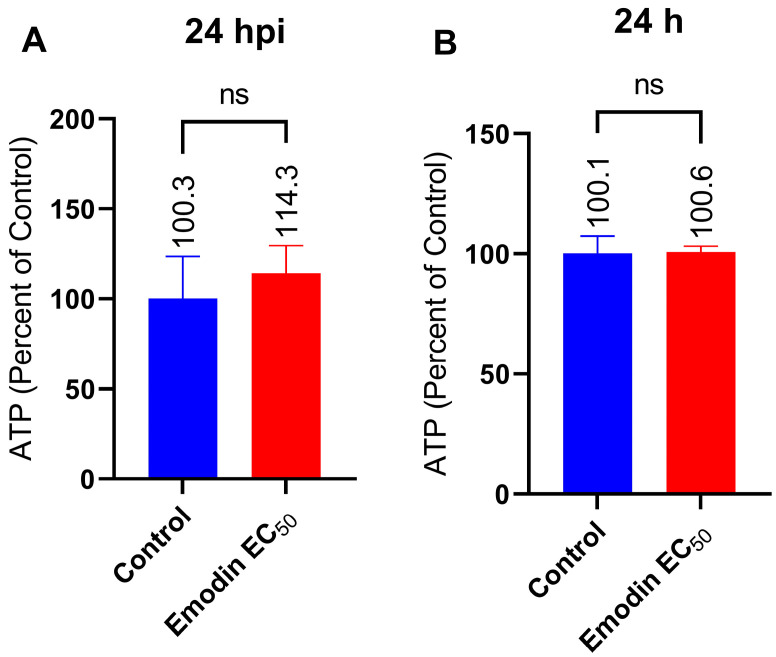
(**A**,**B**) Effect of emodin on mitochondrial toxicity measured as ATP production in the absence and presence of *Toxoplasma* infection. The infected or un-infected HFF monolayers were treated with emodin. After 24 h, the ATP levels were assessed using a Mitochondrial ToxGlo™ assay kit (Promega). The data are means ± standard deviation (SD). The experiment was repeated three times independently. ns—not significant at *p* > 0.05. EC_50_—0.03 µg/mL.

**Figure 12:**
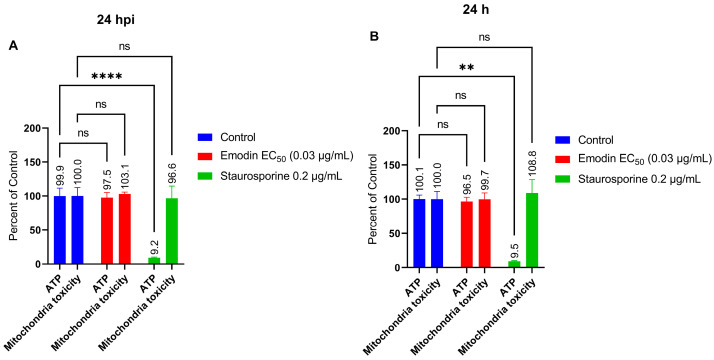
(**A**,**B**) Effect of emodin on mitochondrial membrane integrity measured as cytotoxicity in the absence and presence of *Toxoplasma* infection. The infected or un-infected HFF monolayers were treated with emodin. After 24 h, the mitochondrial membrane integrity and ATP levels were assessed using a Mitochondrial ToxGlo™ assay kit (Promega). The data are means ± standard deviation (SD). The experiment was repeated three times independently. ns—not significant at *p* > 0.05. **—significant at *p* < 0.01 and **** at *p* < 0.0001.

The lack of involvement of ROS or oxidative stress in the anti-parasite action of emodin suggests an alternate mechanism of action. Therefore, we next examined whether emodin affects mitochondria in the absence or presence of *T. gondii* infection. To assess mitochondrial toxicity and monitor ATP production, we employed a commercial assay kit (Mitochondrial ToxGlo™, Promega, Madison, WI, USA). We added emodin to growing HFF monolayers inoculated with fresh parasites and after a 24-h incubation, we measured the mitochondrial ATP production. Emodin did not cause any appreciable change in the ATP level in the presence or absence of *Toxoplasma* infection (Figure 11A,B). Emodin likely did not cause mitochondria toxicity, as ATP production remained unchanged compared to the control. Next, we checked the mitochondria integrity and found that emodin caused no mitochondrial toxicity (Figure 12A,B). Collectively, the ATP and mitochondria toxicity data suggest that emodin is not toxic to mitochondria at the tested doses, whether in the absence or presence of *Toxoplasma* infection. We used staurosporine, a known mitochondrial toxicant, to validate our assay. As expected, staurosporine caused mitochondrial toxicity by reducing (*p* < 0.05) the ATP level, while the mitochondrial integrity remained unchanged compared with the control. This finding not only confirms staurosporine as a mitochondrial toxin, but also supports the validity of our assay.

### 2.7. Caspase 3/7 Activity Is Not Affected by Emodin in the Absence or Presence of Toxoplasma Infection

**Figure 13:**
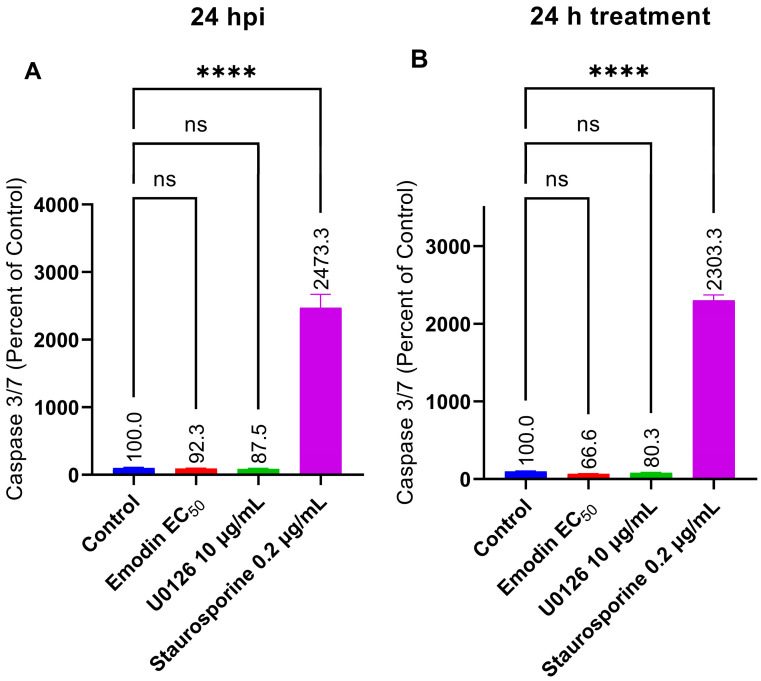
(**A**,**B**) Effect of emodin on caspase 3/7 activity in the absence and presence of *Toxoplasma* infection. The infected or un-infected HFF monolayers were treated with emodin. After 24 h, the caspase activity was determined using a Caspase-Glo^®^ 3/7 assay kit (Promega). The data are means ± standard deviation (SD). The experiment was repeated three times independently. ns—not significant at *p* > 0.05. ****—significant at *p* < 0.0001. EC_50_—0.03 µg/mL.

Given that emodin did not cause oxidative stress and did not affect the mitochondria in the presence or absence of *Toxoplasma* infection, we sought to probe further by investigating other cellular processes, such as apoptosis. We screened for caspase 3/7 activity, which is an important mediator of mitochondrial-driven apoptosis [21]. Our results revealed that caspase 3/7 activity was unaffected (*p* > 0.05) by emodin in the presence or absence of *Toxoplasma* infection when compared to the control (Figure 13A,B). We used staurosporine and U0126, respectively, as a pro-apoptotic and an anti-apoptotic molecule to validate our screening tests [22,23]. As anticipated, staurosporine increased the caspase 3/7 activity significantly (*p* > 0.05) compared to the control, whereas U0126 very slightly (*p* > 0.05) decreased the caspase 3/7 level.

## 3. Discussion

Previously, we demonstrated that emodin has potential as a new anti-parasitic agent [6]. To improve the therapeutic prospects of emodin, we sought to explore its mechanism of action as an anti-parasite agent in an in vitro experimental model of toxoplasmosis. Knowledge of the mechanism of action by way of target identification could aid rational chemistry to modify or synthesize emodin analogues for enhanced selective therapeutic action. In the current study, we tested and confirmed the potential of emodin to restrict the growth of *T. gondii* in vitro. The finding is consistent with our earlier report, which identified new natural molecules that inhibited *T. gondii* growth in vitro [2,6]. Furthermore, our data herein show that the toxicity of emodin toward the parasite was selective and not due to general toxicity.

Our findings also indicate that emodin caused the death of *T. gondii*, both within and outside the host cells. We observed no significant change to the level of parasite growth following the host’s pre-treatment with emodin, suggesting the lack of a host target(s) in the anti-parasitic action. In addition, our data revealed that the action of emodin was not time-dependent, as the anti-parasitic action appeared to peak within the first 24 h, and longer treatment (up to 72 h) did not influence the parasite growth suppression. We further showed that emodin did not affect the parasite invasion of the host cells, thus providing an additional clue as to how it might suppress parasite growth. Parasite invasion was unaffected but under the same conditions, the parasite growth was reduced. Therefore, the anti-parasitic action of emodin involves the inhibition of parasite replication but not invasion. The lytic cycle of *T. gondii* involves the invasion of host cells, intracellular replication, and egress [24].

We checked whether modulation of redox homeostasis or tryptophan metabolism might be involved in the anti-parasite action of emodin. Parasite growth suppression by emodin was not reversed by the addition of l-tryptophan. Previously, we showed that l-tryptophan augmentation could abate the anti-parasitic action of inhibiting molecules including nanoparticles [25], since *Toxoplasma* acquire their l-tryptophan from the host. However, the suppression of parasite growth by emodin was not susceptible to l-tryptophan augmentation, indicating that the host tryptophan pathway is probably not involved in the anti-parasitic action. Together, our findings indicate that the anti-parasite action of emodin lacks a host target, particularly given that the host cell pre-treatment failed to affect parasite growth.

Similarly, augmentation with the antioxidant Trolox failed to reverse the growth suppression caused by emodin. This may indicate that oxidative stress and/or ROS are not involved in the anti-parasite action of emodin. This is not surprising if we consider the fact that emodin possess strong antioxidant properties [13,14,15]. Moreover, consistent with its antioxidant properties, emodin did not significantly affect the global cellular levels of GSH in the absence or presence of *Toxoplasma* infection. To confirm the exclusion of ROS involvement, we assessed global intracellular ROS levels in the absence and presence of the *Toxoplasma* infection. Our data showed that emodin did not influence ROS formation. Interestingly, at the same concentrations and under the same conditions, emodin significantly suppressed parasite viability but not host cell viability. Together, these data suggest the exclusion of oxidative stress and/or ROS production in the anti-parasite action of emodin. Several studies have implicated oxidative stress and/or ROS production in the anti-parasite action of various anti-parasite candidate compounds [24,26,27,28,29]. Studies have shown that ROS formation could be detrimental to parasite growth [26,27]. However, in the present study, our data show no evidence of oxidative stress involvement in the emodin-induced suppression of *Toxoplasma* growth. It appears likely that the mediation of parasite death was through means other than oxidative stress.

Although studies have implicated the mitochondria as a target of several anti-parasite compounds [24,30], the anti-parasite action of emodin did not appear to involve mitochondrial toxicity. The gradient of the mitochondrial inner membrane potential is essential for ATP synthesis. The mitochondria produce the ATP required by the cell. However, if the mitochondrial membrane loses its gradient potential, ATP synthesis is threatened, which causes heat dissipation and energy loss, both of which can be fatal to the cell. Emodin treatment did not cause mitochondrial toxicity. The ATP production remained unchanged compared to the control. Likewise, mitochondrial membrane integrity was unmodified compared to the control. Collectively, the ATP and mitochondrial toxicity data not only support the antioxidant properties of emodin but also suggest that the selective anti-parasitic action of emodin excludes mitochondrial toxicity in the absence or presence of *Toxoplasma* infection. Emodin is a strong antioxidant [13,14,15]; therefore, it is understandable that it may not cause mitochondrial toxicity or deplete the level of ATP, as we observed in the current investigation. Together, our findings indicate that emodin may not be a mitochondrial toxin and that its anti-parasite action might exclude mitochondrial toxicity. These findings are not consistent with our earlier report in which parasite growth inhibitors affected the mitochondrial membrane integrity and caused the death of *T. gondii* [24]. Perhaps emodin behaved differently because it mediates parasite death or growth suppression through other, as yet unidentified, cellular targets. Our findings are also inconsistent with a previous report [31] that showed that emodin induced apoptosis and elevated caspase 3 activity [32]. While the mechanism of action of emodin as a potent anti-parasitic molecule is currently unclear, our data herein indicate that its anti-parasitic action does not involve oxidative stress or apoptosis and the mitochondria. Why emodin does not cause apoptosis or significantly alter caspase 3/7 activity is not yet clear, but we believe cellular physiology may be involved. We showed previously that the bioactive properties of molecules such as curcumin and others vary based on the prevailing physiological environment [29]. For example, there have been reports of emodin causing mitochondria caspase-driven apoptosis in cancer cells [32,33] and of emodin protecting HK-2 renal tubular cells from oxidative stress and apoptotic cell death [10]. Understanding emodin’s mechanism of action as an anti-parasitic agent could serve as a structural basis for the rational design of analogues to improve and enhance selective activity. Recently, a report linked the bioactive properties of emodin to the various substituents on the anthraquinone ring [17]. In addition, a few studies have reported likely cellular targets of emodin in bacteria [34,35]. In one of the studies, emodin ruptured the cell walls, increased the membrane permeability, disrupted the membrane integrity, and caused cytoplasmic vacuolation leading to bacterial cell death [35]. Another study showed that chlorinated emodin induced considerable potassium leakage and cell membrane depolarization, causing harm to the selective permeability of the cell membranes of bacterial cells, which led to bacterial cell death [34]. Additionally, a derivative of emodin has been shown to bind to bacterial DNA and cause DNA condensation [34].

Overall, our findings support and reinforce the pharmacological properties of emodin reported by other researchers. Emodin used as a laxative also has the potential to reduce inflammation [36], besides its various biological functions [37]. Indeed, many commonly used Chinese medicinal herbs contain emodin; for more than 2000 years, emodin has been applied in traditional Chinese medicine, and it is still part of many herbal treatments today [8]. Clinically, anthraquinone-containing medications, such as daunorubicin and mitoxantrone, are components of treatment options to suppress a variety of cancer cells [9]. Moreover, emodin has a wide range of pharmacological properties, including anticancer, hepatoprotective, anti-inflammatory, antimalarial, antioxidant, and antibacterial properties [8,9,17,38].

## 4. Materials and Methods

### 4.1. Materials

Emodin and pyrimethamine were purchased from Selleck Chemicals (Houston, TX, USA), whereas l-tryptophan was from Sigma-Aldrich (St. Louis, MO, USA). Trolox (6-hydroxy-2, 5, 7, 8-tetramethylchroman-2-carboxylic acid) was obtained from Wako Pure Chemicals (Tokyo, Japan), whereas U0126 (MEK inhibitor) was from Santa Cruz Biotechnology Inc. (Dallas, TX, USA). All reagents were of analytical grade and used as supplied unless otherwise indicated.

### 4.2. Evaluation of Emodin for T. gondii Growth Suppressive Activity In Vitro

Unless otherwise stated, we conducted our experiments using a luciferase-reporting *T. gondii* strain (*T. gondii* RH 2F) [39]. We used a luminescence-based assay to assess parasite viability by determining the β-galactosidase (β-gal) activity of the parasite. To maintain the parasite, we employed routine passages in monolayers of human foreskin fibroblast (HFF; ATCC^®^) cells cultivated in Dulbecco’s Modified Eagle Medium (DMEM; Sigma, Tokyo, Japan). The culture medium was supplemented with penicillin and streptomycin (100 U/mL; Lonza™ Biowhittaker™, Slough, UK), GlutaMAX™ (Gibco™ Invitrogen, Waltham, MA, USA), and 10% (*v/v*) fetal calf serum (FCS; Gibco™ Invitrogen, Waltham, MA, USA). To make a parasite suspension, we lysed infected host cells by passing them through a 27-gauge needle and eliminated host debris by filtering the cell lysates through a 5 µm filter. After washing the filtrate with fresh culture media, we used the parasite suspension for in vitro experimental infection analysis as reported elsewhere [24].

### 4.3. Assays for Parasite Invasion and Growth Inhibition

We screened emodin for inhibition of *T. gondii* growth in vitro at various concentrations (between 0.0 and 1000 µg/mL). In addition, we performed a host cytotoxicity assessment at the same concentrations. Based on the results, we performed subsequent experiments with emodin using the EC_50_ (0.03 µg/mL) or EC_90_ (0.05 µg/mL) doses, unless otherwise stated.

We performed the in vitro invasion and growth inhibition assays as reported elsewhere [24]. Briefly, freshly purified parasite suspension was treated with emodin or pyrimethamine (reconstituted in culture media) in growing mammalian host cell monolayers in 96-well optical-bottom plates (Nunc; Fisher Scientific, Pittsburgh, PA, USA). We measured parasite viability after a 72-h incubation in an atmosphere of 37 °C and 5% CO_2_. For the invasion assay, we introduced purified parasite suspension and freshly reconstituted emodin into growing mammalian host monolayers, and incubated the plates for 1 h to permit invasion. We removed the medium and performed a washout to get rid of un-invading parasites. We added new medium to the monolayers and incubated the plates for 72 h in an atmosphere of 37 °C and 5% CO_2_. For the growth inhibition assay, we added purified parasite suspension to growing mammalian host cells, and allowed invasion for 1 h. Following the removal of the media and a thorough washing of the monolayers, we added new medium containing emodin (reconstituted in culture medium) and incubated the plates for 72 h. For all experimental infections, we used an MOI (multiplicity of infection) of 0.2 (ratio of parasite to host cells). The control was medium-treated cells (mock treatment) and the medium-only well was used to account for background signal. To assess parasite viability, we measured β-gal after a 72-h incubation at 37 °C in a 5% CO_2_ atmosphere (Beta-Glo assay kit; Promega, Madison, WI, USA). For all luminescence measurements, we used a GloMax^®^ microplate reader (Promega, Madison, WI, USA). All assays were performed in triplicates and the biological experiments were repeated three times.

### 4.4. Cytotoxicity Assay to Assess Host Cell Viability

We maintained HFF cells in DMEM (Sigma, Tokyo, Japan). The culture medium was supplemented with penicillin and streptomycin (100 U/mL; Lonza™ Biowhittaker™, Slough, UK), GlutaMAX™ (Gibco™ Invitrogen, Waltham, MA, USA), and 10% (*v/v*) fetal calf serum (FCS; Gibco™ Invitrogen, Waltham, MA, USA). After allowing the cells to grow to confluence under normal tissue culture conditions (37 °C and 5% CO_2_), we harvested and seeded the cells at the desired density of 1 × 10^4^ cells per well in 96-well plates (Nunc; Fisher Scientific, Pittsburgh, PA, USA). After a 72-h incubation, we treated the growing monolayers with emodin or pyrimethamine at various concentrations (between 0 and 1000 µg/mL). The control wells contained culture medium without emodin, whereas the medium-only well served to correct for background signal. After a 72-h incubation under normal tissue culture conditions (37 °C and 5% CO_2_), cell viability was determined using a CellTitre-Glo^®^ Luminescent Viability Assay kit (Promega, Madison, WI, USA). All measurements were recorded using a GloMax^®^ microplate reader (Promega, Madison, WI, USA). We performed all assays in triplicates and repeated the biological experiments three times.

### 4.5. Reversibility Assay

To determine whether the emodin parasite growth suppressive effect was reversible, we used a protocol reported elsewhere [24]. Briefly, we added freshly purified parasites and emodin to growing mammalian host monolayers. After incubation for 12 or 24 h in an atmosphere of 37 °C and 5% CO_2_, we removed the medium, washed the monolayers three times, and added fresh medium. After a 24 h incubation, we assessed the parasite viability as described above (Promega, Madison, WI, USA). Parallel to this, we evaluated the parasite growth following each 12- or 24-h incubation in the presence of emodin to compare and assess the capacity of the parasites to recover from the treatment. We performed the biological experiments separately three times.

### 4.6. Assays for Infectivity and Likely Host Cellular Target

We performed this experiment according to a procedure reported elsewhere [24]. For the infectivity assay, we added emodin (reconstituted in culture medium) and freshly purified parasites to a growing monolayer. After a 72-h incubation in an atmosphere of 37 °C in 5% CO_2_, we washed out the treatment thoroughly, used the treated parasites to infect fresh host monolayers, and then incubated the plates for 48 h. We determined parasite viability as described above.

To assess whether the effects on parasite growth were due to host cellular effects, we pre-treated uninfected host cells with either medium or emodin. After a 24- or 48-h incubation at 37 °C in 5% CO_2,_ we washed out the treatments using fresh medium and introduced freshly purified parasites into the pre-treated host cells. We measured the parasite viability after a 24- or 48-h incubation.

In parallel, to check whether emodin had a direct effect on the parasites, we incubated freshly purified parasites with emodin extracellularly. After an 8- or a 12-h incubation in an atmosphere of 37 °C and 5% CO_2_, we assessed parasite viability as described above.

### 4.7. Measurement of Redox Balance

To assess whether treatment with emodin affects redox balance, we measured the intracellular levels of reactive oxygen species (ROS), such as H_2_O_2_ radicals, and an antioxidant molecule (reduced glutathione-GSH), in the absence and presence of *Toxoplasma* infection. Briefly, we treated growing HFF cells with emodin either in the absence or presence of *Toxoplasma* infection. After a 24-h incubation at 37 °C and 5% CO_2_, we determined the intracellular levels of H_2_O_2_ and GSH using commercial assay kits. For the respective H_2_O_2_ and GSH assays, we used ROS-Glo™ H_2_O_2,_ and GSH-Glo™ glutathione kits (Promega, Madison, WI, USA). For ROS assay, we performed a multiplex assay. First, we added the test compounds and initially incubated the plates for 18 h under normal tissue culture conditions. Thereafter, the H_2_O_2_ substrate was added and the plates were incubated for the final 6 h. Afterwards, we transferred 50 µL of the original samples to a new opaque white plate and added ROS-Glo™ detection reagent. We gently shook the plates to mix the contents, incubated the plates for 20 min at room temperature (25 °C) and measured luminescence afterwards. The cells in the original sample plates were used to assay for parasite viability and host cell cytotoxicity, as previously described above. For the GSH assay, we treated the cells and incubated the plates for 24 h under normal tissue culture conditions. Thereafter, we removed the culture medium from the wells and added 100 µL of 1× GSH-Glo™ reagent to each well. We incubated the plates at room temperature (25 °C) for 30 min. Next, we added an equal volume of luciferin detection reagent to each well, mixed contents by gently shaking the plates, and incubated the plates for 15 min at room temperature, before luminescence measurement was performed. Luminescence was read on a GloMax^®^ (Promega, Madison, WI, USA).

### 4.8. Measurement of Mitochondrial Toxicity

To assess mitochondrial toxicity, we used the Mitochondrial ToxGlo™ assay kit (Promega, Madison, WI, USA). We treated growing HFF cells with emodin in the absence or presence of *Toxoplasma* infection. We used staurosporine as a positive drug for mitochondrial toxicity [22]. After a 24-h incubation at 37 °C and 5% CO_2_, we assayed mitochondrial toxicity by fluorescence acquisition on a multi-label plate reader (2030 ARVO™ X5, Version 4.0, Perkin Elmer, Kumamoto, Japan); excitation was at 485 nm and emission was 530 nm. Briefly, after treatment and 24 h incubation under normal tissue culture conditions, we added 20 µL of 5× cytotoxicity reagent to each well. Next, we gently shook the plates to mix the contents and incubated them for 30 min at 37 °C before fluorescence acquisition. For ATP production monitoring, after the fluorescence acquisition, we equilibrated the plates at room temperature (25 °C) for 5–10 min. Next, we added the ATP detection reagent to each well and put the plates on an orbital shaker (500–700 rpm) for 5 min. We determined the ATP level by measuring luminescence on a GloMax^®^ plate reader (Promega, Madison, WI, USA).

### 4.9. Measurement of Caspase 3/7 Activity

To assess caspase 3/7 activity, we used a commercial assay kit (Caspase-Glo^®^ 3/7, Promega, Tokyo, Japan). Briefly, we treated growing HFF cells with emodin in the absence or presence of *Toxoplasma* infection. We used staurosporine and U0126 (MEK inhibitor), respectively, as pro-apoptotic and anti-apoptotic molecules to validate our screening tests [22,23]. After a 24-h incubation at 37 °C and 5% CO_2_, we assayed for caspase 3/7 activity by measuring luminescence on a GloMax^®^ plate reader (Promega, Madison, WI, USA). Briefly, after treatment and a 24-h incubation of plates under normal tissue culture conditions, an equal volume of caspase-Glo^®^ 3/7 reagent was added to each well. The plates were incubated for 1 h at room temperature (25 °C) and the luminescence reading of the samples was recorded afterwards.

### 4.10. Statistical Analysis and Data Presentation

We used one-way and two-way ANOVA (GraphPad Software Inc., San Diego, CA, USA) to analyze the data and Dunnett’s post hoc test to compare among groups with significance taken at *p*-values < 0.05. We presented the data as the mean values ± standard deviation (SD). In addition, we used GraphPad to estimate the concentration of emodin that led to a reduction in parasite and/or host cell viability by 50% (i.e., EC_50_ and/or IC_50_ values, respectively); the curve was fitted with a non-linear regression analysis.

## 5. Conclusions

Efforts to mitigate the global health burden of toxoplasmosis underscore the urgent need for new anti-parasite treatments, and natural bioactive chemicals are crucial sources for new anti-parasitic pharmacophores. Emodin has several pharmacological properties including anti-parasitic activity. However, how it causes *T. gondii* death or growth suppression remains unclear. In the current study, emodin exhibited strong and promising selective action against the parasite versus its host. While our data support parasite targets but not host targets for emodin-mediated parasite growth suppression, our findings suggest that the anti-parasite action of emodin does not involve oxidative stress or ROS production. In addition, our study provides evidence suggesting that the anti-parasite action of emodin excludes mitochondrial toxicity. To our knowledge, the present study is the first report on the action mechanism of emodin as an anti-*Toxoplasma* agent and it provides evidence showing that emodin’s anti-parasitic action does not involve ROS production, oxidative stress, and/or mitochondrial toxicity. Collectively, our findings support the potential of emodin as a promising anti-parasitic agent that warrants further investigation. Our future work will investigate the anti-parasitic potential of emodin in a mouse model.

## Figures and Tables

**Table 1:** Fold activity against *Toxoplasma gondii* versus host cells.

Treatment Group	EC_50_ (RH-2F *Toxoplasma* Tachyzoites) µg/mL	IC_50_ (Human Foreskin Fibroblast—HFF) µg/mL	Selectivity Index (SI)—Ratio of IC_50_ to EC_50_
Emodin	0.03 ± 0.01	8.3 ± 1.3	276
Pyrimethamine	24.0 ± 2.0	62.4 ± 6.0	2.6

The values are the mean of three replicates ± standard deviation (SD). Each experiment was repeated three times independently.

## Data Availability

All data available upon request.

## References

[1] Murata Y., Sugi T., Weiss L.M., Kato K. (2017). Identification of compounds that suppress Toxoplasma gondii tachyzoites and bradyzoites. PLoS ONE.

[2] Adeyemi O.S., Atolani O., Awakan O.J., Olaolu T.D., Nwonuma C.O., Alejolowo O., Otohinoyi D.A., Rotimi D., Owolabi A., Batiha G.E.-S. (2019). Focus: Organelles: In vitro screening to identify anti-*Toxoplasma* compounds and in silico modeling for bioactivities and toxicity. Yale J. Biol. Med..

[3] Attias M., Teixeira D.E., Benchimol M., Vommaro R.C., Crepaldi P.H., De Souza W. (2020). The life-cycle of *Toxoplasma gondii* reviewed using animations. Parasites Vectors.

[4] Schlüter D., Däubener W., Schares G., Groß U., Pleyer U., Lüder C. (2014). Animals are key to human toxoplasmosis. Int. J. Med. Microbiol..

[5] Lu D., Zhang N.Z., Yao Y., Wang T., Hua Q., Zheng X., Cong W., Tan F. (2022). Investigation of Antiparasitic Activity of Two Marine Natural Products, Estradiol Benzoate, and Octyl Gallate, on *Toxoplasma gondii* In Vitro. Front. Pharmacol..

[6] Adeyemi O.S., Sugi T., Han Y., Kato K. (2018). Screening of chemical compound libraries identified new anti-Toxoplasma gondii agents. Parasitol. Res..

[7] Dittmar A.J., Drozda A.A., Blader I.J. (2016). Drug repurposing screening identifies novel compounds that effectively inhibit Toxoplasma gondii growth. Msphere.

[8] Dong X., Fu J., Yin X., Cao S., Li X., Lin L., Huyiligeqi, Ni J. (2016). Emodin: A review of its pharmacology, toxicity and pharmacokinetics. Phytother. Res..

[9] Ji C., Xin G., Duan F., Huang W., Tan T. (2020). Study on the antibacterial activities of emodin derivatives against clinical drug-resistant bacterial strains and their interaction with proteins. Ann. Transl. Med..

[10] Chen H., Huang R.S., Yu X.X., Ye Q., Pan L.L., Shao G.J., Pan J. (2017). Emodin protects against oxidative stress and apoptosis in HK-2 renal tubular epithelial cells after hypoxia/reoxygenation. Exp. Ther. Med..

[11] Yang L., Lin S., Kang Y., Xiang Y., Xu L., Li J., Dai X., Liang G., Huang X., Zhao C. (2019). Rhein sensitizes human pancreatic cancer cells to EGFR inhibitors by inhibiting STAT3 pathway. J. Exp. Clin. Cancer Res..

[12] Janeczko M., Masłyk M., Kubiński K., Golczyk H. (2017). Emodin, a natural inhibitor of protein kinase CK2, suppresses growth, hyphal development, and biofilm formation of Candida albicans. Yeast.

[13] Nam W., Kim S.P., Nam S.H., Friedman M. (2017). Structure-antioxidative and anti-inflammatory activity relationships of purpurin and related anthraquinones in chemical and cell assays. Molecules.

[14] Marković Z., Jeremić S., Marković J.D., Pirković M.S., Amić D. (2016). Influence of structural characteristics of substituents on the antioxidant activity of some anthraquinone derivatives. Comput. Theor. Chem..

[15] Rossi M., Wen K., Caruso F., Belli S. (2020). Emodin scavenging of superoxide radical includes π–π interaction. X-ray crystal structure, hydrodynamic voltammetry and theoretical studies. Antioxidants.

[16] Dalimi A., Delavari M., Ghaffarifar F., Sadraei J. (2015). In vitro and in vivo antileishmanial effects of aloe-emodin on Leishmania major. J. Tradit. Complement. Med..

[17] Friedman M., Xu A., Lee R., Nguyen D.N., Phan T.A., Hamada S.M., Panchel R., Tam C.C., Kim J.H., Cheng L.W. (2020). The inhibitory activity of anthraquinones against pathogenic protozoa, bacteria, and fungi and the relationship to structure. Molecules.

[18] Kim J.-Y., Jung C.-W., Lee W.S., Jeong H.-J., Park M.-J., Jang W.I., Kim E.H. (2022). Emodin coupled with high LET neutron beam—A novel approach to treat on glioblastoma. J. Radiat. Res..

[19] Li X., Chu S., Liu Y., Chen N. (2019). Neuroprotective effects of anthraquinones from rhubarb in central nervous system diseases. Evid.-Based Complement. Altern. Med..

[20] Meier N., Meier B., Peter S., Wolfram E. (2017). In-silico UHPLC method optimization for aglycones in the herbal laxatives Aloe barbadensis Mill., Cassia angustifolia vahl pods, Rhamnus frangula L. Bark, Rhamnus purshianus DC. bark, and Rheum palmatum L. roots. Molecules.

[21] Ponder K.G., Boise L.H. (2019). The prodomain of caspase-3 regulates its own removal and caspase activation. Cell Death Discov..

[22] Cartuche L., Sifaoui I., Cruz D., Reyes-Batlle M., López-Arencibia A., Javier Fernández J., Díaz-Marrero A.R., Piñero J.E., Lorenzo-Morales J. (2019). Staurosporine from Streptomyces sanyensis activates programmed cell death in Acanthamoeba via the mitochondrial pathway and presents low in vitro cytotoxicity levels in a macrophage cell line. Sci. Rep..

[23] You Y., Niu Y., Zhang J., Huang S., Ding P., Sun F., Wang X. (2022). U0126: Not only a MAPK kinase inhibitor. Front. Pharmacol..

[24] Adeyemi O.S., Murata Y., Sugi T., Kato K. (2017). Inorganic nanoparticles kill Toxoplasma gondii via changes in redox status and mitochondrial membrane potential. Int. J. Nanomed..

[25] Adeyemi O.S., Murata Y., Sugi T., Han Y., Kato K. (2017). Modulation of host HIF-1α activity and the tryptophan pathway contributes to the anti-Toxoplasma gondii potential of nanoparticles. Biochem. Biophys. Rep..

[26] Ahmad A., Syed F., Shah A., Khan Z., Tahir K., Khan A.U., Yuan Q. (2015). Silver and gold nanoparticles from Sargentodoxa cuneata: Synthesis, characterization and antileishmanial activity. RSC Adv..

[27] Saini P., Saha S.K., Roy P., Chowdhury P., Babu S.P.S. (2016). Evidence of reactive oxygen species (ROS) mediated apoptosis in Setaria cervi induced by green silver nanoparticles from Acacia auriculiformis at a very low dose. Exp. Parasitol..

[28] Adeyemi O.S., Eseola A.O., Plass W., Kato K., Otuechere C.A., Awakan O.J., Atolani O., Otohinoyi D.A., Elebiyo T.C., Evbuomwan I.O. (2021). The anti-parasite action of imidazole derivatives likely involves oxidative stress but not HIF-1α signaling. Chem.-Biol. Interact..

[29] Adeyemi O.S., Shittu E.O., Akpor O.B., Rotimi D., Batiha G.E.-S. (2020). Silver nanoparticles restrict microbial growth by promoting oxidative stress and DNA damage. EXCLI J..

[30] Charvat R.A., Arrizabalaga G. (2016). Oxidative stress generated during monensin treatment contributes to altered Toxoplasma gondii mitochondrial function. Sci. Rep..

[31] Wang Y., Cui H., Zhou J., Li F., Wang J., Chen M., Liu Q. (2015). Cytotoxicity, DNA damage, and apoptosis induced by titanium dioxide nanoparticles in human non-small cell lung cancer A549 cells. Environ. Sci. Pollut. Res..

[32] Dong X., Ni B., Fu J., Yin X., You L., Leng X., Liang X., Ni J. (2018). Emodin induces apoptosis in human hepatocellular carcinoma HepaRG cells via the mitochondrial caspase-dependent pathway. Oncol. Rep..

[33] Zhang F.-Y., Li R.-Z., Xu C., Fan X.-X., Li J.-X., Meng W.-Y., Wang X.-R., Liang T.-L., Guan X.-X., Pan H.-D. (2022). Emodin induces apoptosis and suppresses non-small-cell lung cancer growth via downregulation of sPLA2-IIa. Phytomedicine.

[34] Duan F., Xin G., Niu H., Huang W. (2017). Chlorinated emodin as a natural antibacterial agent against drug-resistant bacteria through dual influence on bacterial cell membranes and DNA. Sci. Rep..

[35] Li L., Song X., Yin Z., Jia R., Li Z., Zhou X., Zou Y., Li L., Yin L., Yue G. (2016). The antibacterial activity and action mechanism of emodin from Polygonum cuspidatum against Haemophilus parasuis in vitro. Microbiol. Res..

[36] Ambriz-Pérez D.L., Leyva-López N., Gutierrez-Grijalva E.P., Heredia J.B. (2016). Phenolic compounds: Natural alternative in inflammation treatment. A Review. Cogent Food Agric..

[37] Sharma R., Tiku A., Giri A. (2017). Pharmacological properties of emodin—Anthraquinone derivatives. J. Nat. Prod. Resour..

[38] Bayat F., Haghi A.M., Nateghpour M., Rahimi-Esboei B., Foroushani A.R., Amani A., Farivar L., Talaee Z.S., Faryabi A. (2022). Cytotoxicity and Anti-Plasmodium berghei Activity of Emodin Loaded Nanoemulsion. Iran. J. Parasitol..

[39] Ishiwa A., Kobayashi K., Takemae H., Sugi T., Gong H., Recuenco F.C., Murakoshi F., Inomata A., Horimoto T., Kato K. (2013). Effects of dextran sulfates on the acute infection and growth stages of Toxoplasma gondii. Parasitol. Res..

